# Our "Consulting Surgeons."

**Published:** 1900-05-05

**Authors:** 


### The Hospital

A Journal of

The Medical Sciences and Hospital Administration.

Vol. XXVIII.—No. 710.] Edited by Sir Henry Burdett, K.C.B., and Solomon C. Smith, M.D., M.R.C.P. [May 5, 1900.

### Our "Consulting Surgeons."

Amid all the pleasant things which are being said about Sir William MacCormac and Mr. Treves on their return from South Africa—all of which they well deserve—let us not forget that the organisation of the medical arrangements for this war has been the work not of an impromptu civilian service, but of the Army Medical Department. It seems necessary that this should be insisted on since certain writers in the daily Press seem by no means clear upon the subject. Nothing could have been in better form than the graceful words in which Lord Rosebery proposed the toast of the evening, "Sir William MacCormac and Mr. Treves," on the occasion of the dinner given to those gentlemen at the Reform Club last Saturday. It was quite proper that compliments should be the keynote of such a speech, and we join heartily in the praise given to these gentlemen. They did well to go out, and their presence, and that of those who were associated with them, has not only been helpful in the hospitals, but has been an unspeakable comfort to many families among whom the idea was unspeakably horrible that those who were exposed to such unaccustomed risks fighting in South Africa—their husbands, their sons, and all that were most dear—should not in case of accident have the advantage of the "best advice." In every way the presence of consulting surgeons, of high and generally admitted professional position, has been an advantage. But to pretend that the admirable administration and the splendid work done by the medical department is the result of the wisdom of the Government in sending out consulting surgeons to the seat of war is as fulsome as it is absurd.

Lord Rosebery, after stating that our medical and hospital service had been practically perfect, is reported to have said: "Now, that is mainly due to the Government, because the Government have been able to obtain the men, and give the supplies which those men have used. . . . What did the Government do? and how, as I understand, did they achieve this end? They sent the two most eminent men they could think of, and the Government gave them a free hand. (Cheers.) All honour to the Government, and all honour to the men. These are the men whom you are entertaining to-night. (Cheers.) Without a second thought they left their great practice, they left their great connection with this country, and went out for six months, at what sacrifice you can guess, to superintend the hospital and surgical arrangements of our army." This was a graceful speech well conceived in the true spirit of after-dinner oratory, and, like such speeches, not to be analysed too critically. But to be told next morning by *The Times* that "fortunately at a comparatively early period the War Office took the wise course of sending out two most distinguished surgeons to provide for the organisation of the base hospitals and the supervision of the field hospitals, with the result that the wounded in war time have never been more skilfully treated or with smaller loss of life," makes us ask: Is this a true representation of what actually happened? We need hardly say that the position of "consulting surgeon with the field-force" had nothing whatever to do with the organisation of the military hospitals, all of which had fallen to the Army Medical Department. It is to the Royal Army Medical Corps that the credit is due for the perfect medical arrangements which have been characteristic of this as well as of many of our smaller wars. The task has been an enormous one, and when we find it admitted on all hands that it has been well performed, and when we see that no small part of the work of those sent out as consulting surgeons has consisted in chronicling the perfection of the organisation in the midst of which they found themselves, it becomes all the more necessary that, while insisting that the praise for it is due to the Royal Army Medical Corps, we should point out again that for years past this department has been scandalously undermanned, and that its efficient working, which has been the subject of such general praise, and has been of such vital importance to the army at large, has been maintained only at the expense of much personal sacrifice on the part of the officers of the corps.

In regard to the position of the "consulting surgeons," it is probable that the inspectorial functions with which those who first went out to a large extent concerned themselves will now come to an end. The public is satisfied that all is well, and in future the duties of the consultants will be to consult and to operate, rather than to see battles and report on hospitals. As Sir William MacCormac himself has said, the value of consulting surgeons can best be shown by their work at the large base hospitals. During the heat of action, and from the necessarily hasty nature of the surgical work in the field, the opportunities for the employment of special surgical acquirements are less apparent, and the presence of consulting surgeons is of less value than in the large base hospitals, where serious, complicated, and difficult cases are numerous. What, then, we wish to insist upon, while fully joining in the chorus of praise which has greeted our returning consulting surgeons, is that the credit for the perfection of our medical arrangements is due to the Army Medical Department, whose officers, whether doing glorious deeds and braving death on the field of battle or slaving at departmental work in the organisation of great hospitals, have deserved well of their country

